# Construction and application of the emergency resilience capability evaluation scale and model for grassroots medical workers: a cross-sectional study in Guangzhou, China

**DOI:** 10.3389/fpubh.2024.1431237

**Published:** 2024-11-04

**Authors:** Qiaohui Wu, Linjian Wu, Xueqing Liang, Jun Xu, Li Liu, Zhijie Huang, Zhen Ma

**Affiliations:** ^1^Longgang District Central Hospital of Shenzhen, Shenzhen, China; ^2^Guangdong University of Petrochemical Technology, Maoming, China; ^3^Guangzhou Institute of Respiratory Health, Guangzhou, China; ^4^Nanfang Hospital, Southern Medical University, Guangzhou, China; ^5^Dashi Street Community Health Service Center, Guangzhou, China

**Keywords:** attribute analytic hierarchy process, grey correlation analysis, grassroots medical personnel, public health emergency, resilience attribute analytic hierarchy process, resilience

## Abstract

**Objective:**

Public health emergencies pose a serious challenge to the ability of medical personnel to respond. We aimed to establish a scientific and effective resilience capability assessment scale for grassroots medical workers and explore the way to improve resilience.

**Research design:**

Developed a resilience ability assessment scale and applied it on the medical personnel who worked in community health service centers for more than 1 year. Related indicators were identified through literature review and the Delphi method. The established scale was given weight using the attribute analytic hierarchy process, and the evaluation model was built through the grey correlation analysis.

**Principal findings:**

A three-level indicator scale was established, which included three first-level indicators [professional quality (weight 0.346), psychological capital (weight 0.614), and emergency attitude (weight 0.040)]. The second-level indicators had 13 contents and were further subdivided into 46 three-level indicators. The overall resilience ability assessment score of 347 medical workers in Guangzhou was only 0.787. However, steady scientific-exercise habits and relative training can help improve their public health emergency resilience ability (*p* < 0.05).

**Conclusion:**

The establishment of the scale can provide a strategic basis for improving the resilience strength of grass-roots medical personnel. We should encourage this group to form a good habit of exercise, strengthen their crisis awareness by actively organizing theoretical training and practical exercises on public health emergencies, and improve their psychological capital level.

**Patient or public contribution:**

In this study, 347 medical workers from 6 basic medical institutions in Guangzhou participated in the questionnaire.

## Introduction

COVID-19, which emerged in 2019, swept the world in only 4 months. As of February 13, 2023, the cumulative number of confirmed cases of COVID-19 worldwide exceeded 756.58 million, and the cumulative number of deaths was 6.844 million (1), which is the most serious new infectious disease to human society in the past 100 years (2). With the rapid development of the global economy, the public security situation is becoming increasingly severe. As the core subject of the discovery and reporting of public health emergencies, grass-roots medical personnel are an important force in disease prevention and control, and the executive group of the “bottom of the net” work of the health service system. Building a grass-roots team with high resilience in the face of public health emergencies is the core measure of community health service institutions to prevent and respond to public health emergencies.

Foreign research on the ability of medical personnel to deal with public health emergencies mainly focuses on exploring the level of understanding of public health-related laws by medical personnel (3), the ability of emergency preparedness to deal with public health emergencies (4–6) and the level of mastery of professional knowledge of infectious disease prevention and control, research and rescue (7, 8). In China, Zeng Jianbo et al. (9) designed the “Questionnaire on the Response Capacity of Medical Personnel in Medical and Health Institutions to Public Health Emergencies,” which focuses on the evaluation of the response capacity of medical and health institutions to public health emergencies, including the construction of management system, team building, equipment reserves, training drills, public publicity and education, monitoring and early warning and emergency response, without focusing on the response capacity of medical personnel. Wang Zhilong et al. (10), in view of foreign research, designed the “Questionnaire on the Response Ability of Medical Service Organizations in Grassroots Units to Public Emergencies,” focusing on the investigation of medical staff’s medical, epidemiological, legal and other professional knowledge, including general information, basic knowledge of public health emergencies, coping skills, laws, and regulations, etc., The research shows that the coping ability of medical personnel in grass-roots units cannot meet the actual needs. In 2019, Liu Lingyu et al. (11) formed the “core emergency response ability questionnaire of medical staff for major epidemic” with high reliability and validity on the basis of the research on the emergency response ability of medical staff for infectious disease emergencies prepared by Gu Hang (12), Qiao Wenling (13), Kan Ting (14) and other experts. However, the questionnaire is more systematic in terms of prevention ability. The professional ability of medical staff before, during, and after public health emergencies can be evaluated from the three perspectives of preparedness and rescue ability, but the ability of medical staff to respond to public health emergencies cannot be comprehensively evaluated. The research on resilience of different populations were mainly limited to psychological resilience, and there was no systematic research on the assessment system of resilience of medical personnel in the face of public health emergencies.

To sum up, there are not any evaluation systems that can accurately reflect the ability of medical workers’ response to public health emergencies. Some studies can only reflect medical staff’s level of medical professional knowledge. However, the professional nature of medical personnel determines that this population must master corresponding medical knowledge according to their work content, and they have the obligation to master basic medical knowledge and have the strong ability to receive or update their own medical knowledge reserves according to the changes of current events. Medical personnel of different types and different professions have different coping abilities in the face of public health emergencies. Therefore, it is unreasonable to apply a set of capacity evaluation systems barely based on various professional knowledge to evaluate the response ability of medical personnel during public health emergencies.

The Latin word “resilio,” means “bounce to back” (15). This concept originates from physics and characterizes the impact resistance of materials (16). In the 1970s, the ecologist Holling (17) first used the concept of resilience to characterize the ability of the ecosystem to absorb various changes in response to interference when describing the multiple equilibrium states of the ecosystem. Subsequently, the concept of resilience has been more widely used in many fields such as psychology, engineering, ecology, etc. (18). However, until now, there is no accepted definition of resilience in the academic community, and even in the same research field, its meaning is not the same (19). Based on the United Nations definition of resilience (20), this study defines “resilience” as “the ability of medical personnel exposed to public health emergencies to effectively resist, absorb and withstand the impact of disasters, and recover and apply their professional knowledge to protect and repair the necessary infrastructure and functions of their communities,” not limited to “psychological resilience.” Grass-root medical personnel play a major role in the early prevention of public health emergencies, in the middle stage of treatment after the outbreak, or in the late stage of post-disaster reconstruction. In the face of different public health emergencies, medical personnel need to constantly update the corresponding knowledge reserves. We cannot use a single professional knowledge to evaluate the coping ability of the population, but its resilience is the index that best reflects the coping ability of public health emergencies in addition to professional ability.

On the basis of much valuable researches, this study analyzed the shortcomings of the current evaluation systems of medical staff’s response to public health emergencies and established a set of resilience capability evaluation system which was finally applied to prove it can reasonably reflect the response-ability of grass-roots medical staff, providing a scientific reference for gradually improving the response-ability of grass-roots medical staff to public health emergencies.

## Methodology

### The construction progress of the evaluation system

This study began in December 2022, and we searched on CNKI, WIP, Wanfang database, PubMed, Web of Science, the official website of Guangdong Provincial Health Management Commission, and Guangdong Provincial Health Information Network with the keywords of “public health emergencies,” “emergency,” “resilience” and “grass-roots medical workers” etc. Combining the laws and regulations of China and the actual situation of grass-roots medical personnel, and drawing on the experience of population resilience assessment from all over the world, this study had a compilation of 116 indicators at the first step through the methods of literature review and frequency statistics of relevant indicators. Then the indicators which were inconsistent with the actual situation and not practical had been eliminated based on the results of the Delphi method, and the weights of the final 46 indicators were calculated by the attribute analytic hierarchy process to build the resilience evaluation system of grass-roots medical personnel in public health emergencies.

In this study, 25 experts who were selected to participate in the 3 times Delphi consultations (See more basic information about experts in Supplementary Table 1), mainly came from the administrative staff of the community health service institutions in Guangzhou, the epidemiology experts from the School of Public Health of Southern Medical University and the School of Public Health of Guangzhou Medical University, and the public health management professors of the School of Health Management of the Southern Medical University. After the 3 Delphi expert consultations, we have finally determined 46 evaluation indicators. At last, the experts were additionally invited to evaluate the importance of each indicator according to the Saaty1-9 scale sheet (as shown in Supplementary Table 2) to create the comparison matrix B .

### Participants and data collection

In the period of 2022–2023, this constructed system was final applied investigate the medical staff of 6 community health service centers in Guangzhou, Guangdong Province. The surveys began in September 2022 and ended in October 2023. Data were collected using a combination of online and paper questionnaires. While assessing the emergency resilience of medical workers in the community health service institution, we collected some basic information about them, including gender, years of work, job nature, major during education, the status of obtaining relevant medical certificates (Chinese physician qualification certificate/Red Cross ambulance certificate/AHA Certificate/ITLS Certificate, etc), exercise habits, and their degree of recognition about the arrangements of their work during the period of public health emergencies, and so on. At the end of the study, 417 workers were participated, and 70 workers’ records were excluded. Patients and the public were not involved in the design of this study.

### Statistical analysis

#### Analytic hierarchy process (AHP)

AHP, as a qualitative and multi-objective decision analysis research method, is widely used in the field of comprehensive evaluation of multiple indicators (21). By using this method, the evaluation results of the evaluators can be quantified, and the effectiveness and reliability of the evaluation can be improved, especially in solving complex problems such as multiple indicators and multiple objectives. Compared with the AHP, the Attribute Hierarchy Model (AHM) created by Professor Qiansheng Cheng (22) has the same decision-making process, but AHM does not require the eigenvector of the judgment matrix, only needs and operations. AHP requires consistency, while AHM does not require consistency. Since the AHM is reasonable and simple, and it has been widely used in China (23, 24), we used it to formulate weights for the evaluation system.

#### Grey relation analysis (GRA)

In the process of evaluating the resilience of grass-roots medical personnel, the advantages and disadvantages of some indicators cannot be unified and specifically reflected. As a result, when evaluating, it is frequently impossible to make accurate judgments on these indicators and thus obtain clear evaluation results. Therefore, this study conducted the grey correlation analysis based on the whitening weight function to quantify the advantages and disadvantages of the indicators. This method takes the whitening of the system as the core to predict and control the current situation and development trend of the system. By constructing the whitening weight function, the whitening law of the grey hazy concentration in the system can be accurately expressed mathematically.

#### Grey system theory

Professor Julong Deng, a Chinese scholar, put forward the grey system theory in 1982, which is to realize the accurate description and understanding of certain things through the research and calculation of some existing information (25). The grey correlation analysis of indicators is the most widely used part of the grey system theory. It is a method to find the importance of indicators according to their similarity. GRA is usually used to solve multi-attribute decision-making problems by integrating various performance attribute values into one value (26). In the study, the information in the white system is known, the system with unknown information is the black system, and the system with incomplete or unclear information is the grey system. In fact, the grey system is a tool for evaluation in the case of uncertain or incomplete data.

#### Grey whitening weight function

The whitening weight function is a functional expression of the value of each element in the gray number (gray category), which quantitatively describes the degree to which a gray number (i.e., data index) determines each value in its value area. When dealing with practical problems, researchers use the data information of the clustering object to construct the whitening weight function based on the known information. Although there is no unified formula, the starting point and ending point of the curve have the meaning of the clustering object. The value of each turning point is the key to the whitening weight function. Due to its simpler form and more intuitive graphics, it is widely used in comprehensive evaluation. The commonly used whitening weight function has four basic forms: typical whitening weight function, upper limit measure whitening weight function, lower limit measure whitening weight function, and moderate measure whitening weight function.

This study established an evaluation model by combining attribute hierarchy analysis and grey correlation analysis. According to the established comprehensive evaluation system of resilience, the resilience evaluation results of grass-roots medical personnel in the face of public health emergencies were divided into four gray categories: “excellent,” “good,” “medium” and “poor.” According to the specific conditions of each level indicators, three upper limit measure whitening weight functions and one lower limit measure whitening weight function were constructed, so four kinds of grey evaluation coefficients need to be solved, respectively. Based on the weight of each indicator, we performed the operation steps to determine the grey evaluation parameters of each indicator.

## Results

### Establish the resilience evaluation system of grass-roots medical personnel based on Delphi method and assign the indicators’ weights through AHP

This study explored the resilience level of medical staff in public health emergencies from three aspects: professional quality, psychological capital and emergency attitude. The resilience evaluation system of grassroots medical personnel in public health emergencies was divided into three levels, and the consistency coefficient Kendall’s W of the 3 times Delphi consultations can be seen in Table 1.

**Table 1:** Consistency coefficient for three rounds of Delphi consultations.

Classification	Round 1			Round 2			Round 3		
	The number of indicator	Kendall’s W	*p*	The number of indicator	Kendall’s W	*p*	The number of indicator	Kendall’s W	*p*
Professional quality	31	0.418	0.00	17	0.538	0.00	17	0.511	0.00
Psychological capital	41	0.397	0.00	34	0.351	0.00	34	0.330	0.00
Emergency attitude	44	0.305	0.00	29	0.364	0.00	29	0.372	0.00
Total	116	0.372	0.00	80	0.409	0.00	80	0.394	0.00

The first layer included three first-level indicators, including professional quality, psychological capital and emergency attitude. The second layer was composed of 13 s-level indicators, and the third layer was final composed of 46 third-level indicators, which reflected the resilience level of grass-roots medical personnel in various aspects in the face of public health emergencies from the aspects of organization and coordination ability, training attitude and prevention awareness, and more comprehensively covered all internal factors that might affect the resilience of the population. For the convenience of expression, third-level indicators were labeled as $\chi_{hij}$ . The resilience evaluation system with assigned weights is shown in Table 2. According to the pairwise comparison matrix of indicators at all levels, the weights of indicators at all levels were determined by using the AHM. The weight of the first-level indicator “Professional quality” of grass-roots medical personnel is 0.346, and the subordinate second-level indicator with the highest weight is “Communication skills” (0.465); Among the first-level indicators, “Psychological capital” has the highest weight of 0.614, and its subordinate second-level indicators have the highest weight of “Effectiveness” (0.445); The first-level indicator “Emergency attitude” has the lowest weight (0.040), and its subordinate second-level indicator “Awareness of prevention” (0.363) has the highest weight.

**Table 2:** Evaluation index system and weight value of resilience of grassroots medical personnel in the face of public health emergencies.

First-level indicators	Weights of first-level indicators	Second-level indicators	Weights of second-level indicators	Third-level indicators	Weights of third-level indicators
Professional quality x1	0.346	Communication skills x11	0.465	I can communicate well with the residents in dialect (for example in Guangdong province: Cantonese). x111	0.200
				I can assist the unit in the documentation and publicity work (such as the writing of the official account of the unit, propaganda and education documents, etc.). x112	0.396
				At work, I can express academic ideas accurately, scientifically and rigorously, and exchange research results. X113	0.404
		Social mobilization x12	0.035	I am will to have the initiative to understand the public health situation at the global, national, regional and community levels. x121	0.286
				I would like to find community partners to carry out actions to promote the health of the residents. x122	0.377
				I will pay close attention to current public health policies and seek opportunities to participate in activities that improve them. X123	0.337
		Teamwork x13	0.185	When performing tasks, I prefer team tasks where I can work with others. x131	0.275
				When carrying out emergency rescue tasks with the team, I will actively learn task-related information and actively share with members. x132	0.725
		Organization and coordination skills x14	0.315	During public health operations, I can quickly adjust myself to align my work status with the organization’s activities. x141	0.512
				I can encourage others to participate in action so as to promote internal collaboration. x142	0.488
Psychological capital x2	0.614	Optimism x21	0.039	I always see the bright side of things in my work. x211	0.275
				I am optimistic about what will happen to my job in the future. x212	0.259
				In the face of public health events such as the COVID-19 pandemic, I always believe that “there is a light at the end of the tunnel, do not be pessimistic.” x213	0.466
			Toughness x22	0.198	I believe I can work through any challenge I have at work. x221	0.220		
				I can do it on my own if I have to do hard work alone. x222	0.254		
				I usually take stress at work in stride. x223	0.063		
				Even in the face of public health outbreaks, I am able to survive difficult times at work. x224	0.230		
				In my current job, I feel like I can juggle many things at once. x225	0.234		
		Hope x23	0.318	If I find myself stuck at work, I can think of many ways to get out of it. x231	0.313		
				At present, I am full of energy to complete my work goals. x232	0.097		
				There are many solutions to any problem. x233	0.137		
				At the moment I am quite satisfied with what I have achieved in my work. x234	0.100		
				I can think of a number of ways to achieve my current workday goals. x235	0.192		
				At the moment, I am achieving the work goals I set for myself. x236	0.161		
		Effectiveness x24	0.445	I believe I can analyze long-term problems and find solutions. x241	0.163		
				In meetings, I am confident in stating things that are within the scope of my job. x242	0.065		
				I think I can improve the general well-being in the community which I work for. x243	0.184		
				Within the scope of my job, I believe I can help set goals/objectives. x244	0.137		
				I believe in my ability to connect with people outside my working department to solve some related problems. x245	0.177		
				If I were in the scene of a public health emergency, I believe I would be able to calmly analyze the situation and work on the front lines as soon as possible. x246	0.273
Emergency attitude x3	0.040	Disaster relief expectation x31	0.277	If I have chance, I am willing to participate in the rescue work of public health emergencies. x311	0.277
				If I have chance, I am willing to assist in the disinfection of the epidemic area. x312	0.218
				During the public health emergency, I would be willing to receive psychological training to provide the psychological care and interventions that patients or community residents need. x313	0.235
				As a grass-roots medical worker, if I realize that there is a crisis of public health events such as infectious diseases, I will do all in my power to send a protective warning to the outside world. x314	0.269
		Trained attitude x32	0.191	I am willing to learn more about public health emergencies x321	0.197
				It is necessary to carry out public health emergency related courses and training. x322	0.259
				It is necessary to carry out emergency drills for public health emergencies. X323	0.246
				It is important to master the basic knowledge of public health emergencies. x324	0.297
		Mental reaction x33	0.040	I am terrified of a public health emergency. X331	0.533
				Performing tasks related to public health emergencies such as daily epidemic prevention will make me feel stressed. x332	0.467
		Awareness of prevention x34	0.363	It is necessary to equip the household with emergency medicine kit (containing disinfectant, mask, anti-inflammatory drugs, anti-allergic drugs and other items), fire extinguisher and other basic emergency materials. x341	0.256
				I am willing to assist community residents to carry out preventive vaccination and preventive medication. x342	0.336
				I am willing to actively communicate with community residents and carry out health education on public health emergencies. x343	0.409
		Self-evaluation x35	0.129	I am familiar with public health emergencies. x351	0.356
				I will pay attention to the epidemic situation of local infectious diseases actively. x352	0.299
				I will follow the World Health Organization’s reports on various public health emergencies. x353	0.345

### The resilience of medical workers from 6 community health service institutions in Guangzhou, Guangdong Province

We applied the established resilience assessment scale for grass-roots medical personnel in public health emergencies to conduct a survey of medical personnel in 6 community health service institutions in Panyu District, Tianhe District, Baiyun District, Nansha District, Guangzhou, Guangdong Province (see the relevant questionnaire in Supplementary materials named “Resilience-Self-Evaluation Scale for Grassroots Medical Workers”). A total of 417 questionnaires were distributed in this study, and 347 were effectively recovered, with a recovery rate of 83.2%.

The overall assessment score of emergency resilience of medical staff in the community health service center was 0.749 (0.060), which is in the medium range. Among the first-level indicators, their “Professional quality” (0.752 ± 0.0045) has the highest score, with its subordinate indicator “Teamwork” (0.754 ± 0.0047) having the highest score among all the second-level indicators. Their “Psychological capital” was the lowest score (0.736 ± 0.0057). In the whole second-level indicators, the score of “Mental reaction” (0.698 ± 0.0047) was the lowest. For more details, please see Table 3.

**Table 3:** The evaluation score of each level indicator through GRA model analysis.

First-level indicators	Median (QR)	Second-level indicators	Median (QR)	Third-level indicators	Median (QR)
Professional quality x1	0.765 (0.070)	Communication skills x11	0.765 (0.070)	I can communicate well with the residents in dialect (for example in Guangdong province: Cantonese). x111	0.765 (0.076)
				I can assist the unit in the documentation and publicity work (such as the writing of the official account of the unit, propaganda and education documents, etc.). x112	0.765 (0.076)
				At work, I can express academic ideas accurately, scientifically and rigorously, and exchange research results. x113	0.765 (0.076)
		Social mobilization x12	0.765 (0.076)	I am will to have the initiative to understand the public health situation at the global, national, regional and community levels. x121	0.765 (0.076)
				I would like to find community partners to carry out actions to promote the health of the residents. x122	0.765 (0.076)
				I will pay close attention to current public health policies and seek opportunities to participate in activities that improve them. X123	0.765 (0.076)
		Teamwork x13	0.765 (0.076)	When performing tasks, I prefer team tasks where I can work with others. x131	0.765 (0.076)
				When carrying out emergency rescue tasks with the team, I will actively learn task-related information and actively share with members. x132	0.765 (0.076)
		Organization and coordination skills x14	0.765 (0.076)	During public health operations, I can quickly adjust myself to align my work status with the organization’s activities. x141	0.765 (0.076)
				I can encourage others to participate in action so as to promote internal collaboration. x142	0.765 (0.076)
Psychological capital x2	0.748 (0.060)	Optimism x21	0.759 (0.083)	I always see the bright side of things in my work. x211	0.724 (0.101)
					I am optimistic about what will happen to my job in the future. x212	0.765 (0.126)		
		In the face of public health events such as the COVID-19 pandemic, I always believe that “there is a light at the end of the tunnel, do not be pessimistic.” x213			0.765 (0.076)		
		Toughness x22	0.744 (0.073)	I believe I can work through any challenge I have at work. x221	0.765 (0.076)		
				I can do it on my own if I have to do hard work alone. x222	0.765 (0.101)		
				I usually take stress at work in stride. x223	0.724 (0.091)		
				Even in the face of public health outbreaks, I am able to survive difficult times at work. x224	0.765 (0.101)		
				In my current job, I feel like I can juggle many things at once. x225	0.724 (0.126)		
		Hope x23	0.741 (0.076)	If I find myself stuck at work, I can think of many ways to get out of it. x231	0.724 (0.066)		
				At present, I am full of energy to complete my work goals. x232	0.765 (0.076)		
				There are many solutions to any problem. x233	0.765 (0.076)		
				At the moment I am quite satisfied with what I have achieved in my work. x234	0.724 (0.091)		
				I can think of a number of ways to achieve my current workday goals. x235	0.724 (0.076)		
				At the moment, I am achieving the work goals I set for myself. x236	0.765 (0.126)		
		Effectiveness x24	0.745 (0.057)	I believe I can analyze long-term problems and find solutions. x241	0.724 (0.084)		
				In meetings, I am confident in stating things that are within the scope of my job. x242	0.765 (0.108)		
				I think I can improve the general well-being in the community which I work for. x243	0.765 (0.076)		
				Within the scope of my job, I believe I can help set goals/objectives. x244	0.724 (0.076)		
				I believe in my ability to connect with people outside my working department to solve some related problems. x245	0.765 (0.076)		
				If I were in the scene of a public health emergency, I believe I would be able to calmly analyze the situation and work on the front lines as soon as possible. x246	0.724 (0.076)
Emergency attitude x3	0.759 (0.070)	Disaster relief expectation x31	0.765 (0.076)	If I have chance, I am willing to participate in the rescue work of public health emergencies. x311	0.765 (0.076)
				If I have chance, I am willing to assist in the disinfection of the epidemic area. x312	0.765 (0.076)
				During the public health emergency, I would be willing to receive psychological training to provide the psychological care and interventions that patients or community residents need. x313	0.765 (0.076)
				As a grass-roots medical worker, if I realize that there is a crisis of public health events such as infectious diseases, I will do all in my power to send a protective warning to the outside world. x314	0.765 (0.076)
		Trained attitude x32	0.765 (0.076)	I am willing to learn more about public health emergencies x321	0.765 (0.076)
				It is necessary to carry out public health emergency related courses and training. x322	0.765 (0.076)
				It is necessary to carry out emergency drills for public health emergencies. X323	0.765 (0.076)
				It is important to master the basic knowledge of public health emergencies. x324	0.765 (0.076)
		Mental reaction x33	0.689 (0.064)	I am terrified of a public health emergency. X331	0.674 (0.102)
				Performing tasks related to public health emergencies such as daily epidemic prevention will make me feel stressed. x332	0.724 (0.126)
		Awareness of prevention x34	0.765 (0.076)	It is necessary to equip the household with emergency medicine kit (containing disinfectant, mask, anti-inflammatory drugs, anti-allergic drugs and other items), fire extinguisher and other basic emergency materials. x341	0.765 (0.076)
				I am willing to assist community residents to carry out preventive vaccination and preventive medication. x342	0.765 (0.076)
				I am willing to actively communicate with community residents and carry out health education on public health emergencies. x343	0.765 (0.076)
		Self-evaluation x35	0.738 (0.069)	I am familiar with public health emergencies. x351	0.724 (0.091)
				I will pay attention to the epidemic situation of local infectious diseases actively. x352	0.765 (0.076)
				I will follow the World Health Organization’s reports on various public health emergencies. x353	0.765 (0.076)

A full score is 1, a score below 0.4 is poor, a score in (0.4, 0.6] is medium, a score in (0.6, 0.8] is good, and a score in (0.8, 1.0] is excellent.

### Exploration of the influencing factors of grassroots medical personnel’s resilience in public health emergencies

We explored the relationship between some of the potential influencing factors and the assessed resilience scores, and the results were shown in Supplementary Table 4. We found that grassroots medical personnel with different years of work had different scores of emergency attitude (*p* = 0.048), enthusiasm (*p* = 0.036), and prevention awareness (*p* = 0.031) in emergency attitude, teamwork ability (*p* = 0.013) and organization and coordination ability (*p* = 0.021) in professional quality. The results of the comparison between groups showed that the differences in the above-related indicators between the grassroots medical personnel with years of work ranging from 11 to 15 and the grassroots medical personnel with years of work ranging from 5 to 10 were statistically significant (*p* < 0.05), and the grassroots medical personnel with working years ranging from 11 to 15 had the highest score. The work nature and status of obtaining relevant medical certificates had no statistical significance on the resilience indicators of grassroots medical personnel. It is worth noting that for the indicator numbered x231, the score of grassroots medical personnel educated in “Clinical medicine” [0.765 (0.076)] was higher than that of primary medical staff educated in “Public health and preventive medicine” [0.724 (0.084)], and the difference was statistically significant (*p* < 0.05).

Grassroots medical personnel with different exercise habits have different total scores of emergency resilience assessment (*p* = 0.040), and their professional literacy scores (*p* = 0.050) and communication ability scores (*p* = 0.005) in professional literacy are also different. Grassroots medical personnel with stable exercise habits tend to have higher scores in various emergency resilience assessment indicators than those who do not like exercise, and partial results of the pin-two comparison showed that the difference was statistically significant (*p* < 0.05).

Grassroots medical personnel who have participated in emergency theoretical training were better at communicating with community residents [0.765 (0.045), *p* = 0.018] and were more inclined to cooperate with others when performing tasks [0.765 (0.076), *p* = 0.005]. Moreover, the scores of grassroots medical personnel participating in emergency drills were significantly higher in both professional literacy [0.765 (0.058), *p* = 0.002] and emergency attitude [0.762 (0.059), *p* = 0.048] than those of grassroots medical personnel not participating in actual emergency drills, and the differences were statistically significant (*p* < 0.05).

## Discussion

An emergency resilience ability assessment system has been created for grass-roots medical personnel. It is based on four principles: scientificity, systematism, operability, and applicability. The system is made up of three parts: professional quality, psychological capital, and emergency attitude. The first-level indicator with the highest weight coefficient is “psychological capital.”

Professional quality refers to the internal norms and requirements of the profession and is the comprehensive quality displayed in the course of the profession, including professional ethics, professional skills, professional behavior, professional style, and professional awareness (27). In the resilience assessment system built in this study, the professional quality of grassroots medical personnel focuses on the basic abilities, qualities, and accomplishments that the population should have in response to public health emergencies in the process of basic medical and health work, including communication and communication ability, social mobilization ability, team cooperation ability, organization, and coordination. Grassroots medical workers are the “health gatekeepers” of the people. Their work focuses not only on the sick but also on the healthy. They should not only be able to cure common diseases and frequently-occurring diseases, but they should also have public health knowledge and strong communication skills to raise people’s health awareness. Professional quality is essential for professionals to use their professional knowledge to effectively solve relevant responsibilities. Grassroots medical personnel of high professional quality who are exposed to public health emergencies can use their professional knowledge more effectively to protect and repair the necessary infrastructure and functions of their communities, which is a fundamental component of resilience assessment.

Seligman, an American psychologist, first proposed the concept of psychological capital in the 1990s, pointing out that a high level of psychological capital can effectively reduce work pressure and alleviate the adverse effects caused by individual negative emotions (28). The term psychological capital is then commonly used in sociology, economics, and investment. People currently accept the definition of psychological capital proposed by Luthans as the positive psychological state of individuals in the process of growth and development that can guide individuals to generate positive cognition and thus influence individuals to generate positive behavior (29). The emphasis is on the fact that it can be enhanced via acquired training. It is a significant resource in addition to social and human capital, and it has four aspects. That is the optimism-positive expectation of action results or positive interpretation style of events; Resilience - the ability to self-adjust, face positively, bear bravely, recover quickly, and achieve success through alternative ways when in trouble; Hope - to make unremitting efforts to achieve the goal and to flexibly change to new methods or approaches when necessary; Self-efficacy - the confidence to have the courage to undertake challenging tasks, and to be able to stimulate their motivation and achieve success through sufficient efforts (30). This study investigated “psychological capital” in terms of the four dimensions mentioned above. The psychology of medical staff with varying levels of psychological capital has varying degrees of influence in primary care. Higher levels of psychological capital can improve medical staff’s creativity, job satisfaction, and job performance while decreasing work pressure, turnover rate, and job burnout, and vice versa. This is the foundation of medical staff resilience assessment in public health emergencies.

This study’s emergency attitude refers to grassroots medical personnel’s belief and attitude toward responding to public health emergencies based on their knowledge, information, and learning. According to the well-known “knowledge, attitude, and practice” (KAP) theory, “knowledge” refers to knowledge, information, and learning, “belief” refers to attitude and belief, and “action” refers to action and behavior (31). In terms of the theory, knowledge is the foundation for developing correct attitudes and positive beliefs, and thus changing behavior. Beliefs and attitudes drive behavior change, and people’s behavior is influenced by their knowledge and attitudes (32). As a result, grass-roots medical personnel’s belief and attitude in emergency response is at the heart of their ability to respond to public health emergencies by their abilities and is an important component of medical personnel’s resilience assessment in public health emergencies.

To summarize, the resilience evaluation system developed in this study is scientific and authoritative, and it is a comprehensive evaluation system capable of reflecting the resilience of grassroots medical personnel in public health emergencies comprehensively and objectively. China’s public health service governance capability was highlighted at the start of the COVID-19 outbreak in 2020, and it played a significant role in the prevention and control of major diseases, which was widely recognized around the world. Grassroots medical personnel are critical in protecting residents’ physical and mental health before, during, and after public health emergencies. Active guidance to residents is an important measure to improve community resilience in the face of public health emergencies, as well as an important way for community medical personnel to participate in mitigating the harm of public health emergencies to the physical and mental health of community residents. It is necessary to strengthen the resilience of grassroots medical personnel to respond to public health emergencies and to improve community public health governance capacity to prevent large-scale deterioration of public health emergencies, promote the development of health endeavors, and improve residents’ health. At the moment, many countries’ medical and health systems are still “profit-oriented, “falling into a vicious circle of “emphasizing medical treatment while neglecting prevention.” There are numerous issues in grassroots medicine: (1) The emergency team is not sound, and the strength of the medical staff is not fully exploited; (2) There are blind spots in the information communication between the medical staff in the grassroots medical institutions; (3) The crisis awareness of grassroots medical personnel is weak (33, 34). As a result, we must urgently establish and improve the resilience assessment system of grassroots medical workers, identify this population’s underserved links in response to public health emergencies, improve the scientific and reasonable basis for building a strong force to prevent and respond to public health emergencies and provide a strong reference for future talent training planning in the world’s grass-roots medical institutions.

### How to enhance the resilience capability of grassroots medical personnel in face of public health emergencies: we should pay attention to cultivating the exercise habits of this population

The results of this study on the resilience ability of medical staff in 6 community health service institutions in Guangzhou show that the overall level of grassroots medical personnel was at a medium level and needs to be improved. Based on the results of the exploration of potential influencing factors, we propose improving the resilience capability of grassroots medical personnel in the face of public health emergencies from the following three aspects. Firstly, primary medical institutions should actively and regularly organize sports-related activities for their staff, which can cultivate their sports preferences, encourage them to develop stable exercise habits and promote communication among colleagues in various departments. Our research results preliminarily showed that grassroots medical personnel with stable exercise habits had better scores in various resilience assessments than those who occasionally exercise or dislike exercise, especially in indicators of professional literacy. Furthermore, numerous studies have concluded that consistent exercise can reduce long-term joint stress, “resist” age-related physiological deterioration, improve health outcomes, and promote cognitive function and psychological well-being (35, 36). Therefore, actively advocating stable and reasonable sports for grassroots medical personnel can greatly promote the population’s resilience in the face of public health emergencies, and is conducive to improving the population’s work efficiency. Secondly, while psychological capital has the highest weight in the overall emergency resilience assessment system, the research findings revealed that the score of psychological capital of grassroots medical personnel was the lowest among all first-level indicators, indicating that the psychological construction of grassroots medical personnel needs to be strengthened. Accordingly, it is critical to establish and improve a professional psychological counseling system for medical staff and prioritize psychological health education for grassroots medical personnel to cultivate this group’s courage to take on difficult tasks and inspire their work motivation. Government agencies should support the development of psychological counseling platforms for various professions and encourage employees from all walks of life to have strong psychological capital to improve their work resilience and productivity. Thirdly, this study showed that grassroots medical workers who have participated in public health emergency drills had higher professional literacy and more positive emergency response attitudes than those who have not participated in the drills while participating in theoretical training on public health emergencies could promote individual team collaboration expectations and communication skills. This indicates that we should closely combine and enhance both theoretical training and practical exercises for public health emergencies, which not only increase their crisis awareness to foster a positive emergency response attitude but also improve their resilience in crisis management. A good emergency attitude enables us to make proper preparations for the prevention and control of material reserves, public awareness and education, emergency management, and other areas, as well as respond effectively to the crisis.

### Limitations

The assessment scale can be widely used to assess the resilience ability of grassroots medical personnel. If it is promoted to other levels of medical staff or other occupational groups, the above indicators and weights need to be revised and improved by the group’s characteristics. The factors influencing the resilience capability of grassroots medical personnel in this study needs to be further explored by increasing the research content and further expanding the sample size.

## Conclusion

This assessment scale applied to grassroots medical personnel can scientifically and systematically assess the population’s resilience ability in face of public health emergencies. Unlike previous evaluation studies that only examined medical workers’ level of medical knowledge mastery, this study conducted a comprehensive assessment of their resilience based on the premise that as medical workers, they must master corresponding medical knowledge based on their professional characteristics. The evaluation system is highly practicable, and its applied preliminary results showed that the overall toughness of primary medical staff in Guangzhou is at a medium level, particularly the psychological capital in the toughness evaluation, which needs to be improved through active measures such as encouraging primary medical staff to develop good exercise habits, and regular training and drills can help improve the resilience of grassroots medical workers in the face of public health emergencies.

## Key messages

### What is already known on this topic

Currently, research on the emergency response capability of medical personnel focused on their mastery of medical professional knowledge, and the establishment of emergency response capability evaluation systems frequently involved the simple use of highly subjective expert consultation methods. No research had been conducted to scientifically and systematically establish a set of emergency resilience assessment systems applicable to grassroots medical personnel from the perspective of assessing medical professional knowledge that medical personnel are obligated to master, nor had any research been conducted to establish a set of emergency resilience assessment models combining attribute hierarchical analysis and gray correlation analysis, nor had any research been conducted to explore the influencing factors that affect the emergency resilience of grassroots medical personnel.

### What this study adds

An emergency resilience assessment system for grassroots medical personnel in public health emergencies was established based on the above literature researches, expert consultation, and attribute analysis methods. The evaluation scale was applied to grassroots medical workers in communities in Guangzhou. To begin, it built a three-in-one emergency resilience assessment system based on identifying important populations for emergency response to public health emergencies; Second, it employed scientifically the gray correlation analysis approach to conclude the scores of numerous indicators completely. Lastly, preliminary research was conducted on the possible effect variables of this group’s resilience capability based on a Cross-sectional survey in Guangzhou, China.

### How this study might affect research, practice or policy

The study created a scientific and systematic evaluation system for grassroots medical personnel’s resilience ability in response to public health emergencies, which has both practical and referential value. The application of this evaluation system in communities in Guangzhou reveals that the emergency resilience ability of grassroots medical workers in Guangzhou is at a medium level, particularly the “psychological capital, “and that effective measures are required to improve it, such as encouraging grassroots medical staff to cultivate reasonable and stable exercise habits, closely combining theoretical training on the theme of “public health emergencies” with practical exercises, and so on.

## Reflexivity statement

The authors include three females and four males and span multiple levels of seniority. While four of the authors specialize in health policy management, the second is a data analysis researcher, and the third and sixth are doctors from different medical authorities. All authors have extensive experience conducting public health researches and policy studies.

## Data Availability

The raw data supporting the conclusions of this article will be made available by the authors, without undue reservation.
