# Supplementary material for: Construction and application of the emergency resilience capability evaluation scale and model for grassroots medical workers: a cross-sectional study in Guangzhou, China
Source: Front Public Health. 2024 Nov 4;12:1431237. doi: 10.3389/fpubh.2024.1431237 (PMC11572528; doi:10.3389/fpubh.2024.1431237)
Supplement: Supplementary file 1 [file Table_1.DOCX]

**Supplementary Table 1:** Basic information of experts participating in three rounds of Delphi expert consultation

|  | Round 1 | Proportion (%) | Round 2 | Proportion (%) | Round 3 | Proportion (%) |
| --- | --- | --- | --- | --- | --- | --- |
| Gender |  |  |  |  |  |  |
| Male | 5 | 62.50% | 17 | 77.27% | 16 | 76.19% |
| Female | 3 | 37.50% | 5 | 22.73% | 5 | 23.81% |
| Age |  |  |  |  |  |  |
| 30～ | 6 | 75.00% | 10 | 45.45% | 9 | 42.86% |
| 40～ | 2 | 25.00% | 9 | 40.91% | 9 | 42.86% |
| 50～ | 0 | 0.00% | 3 | 22.73% | 3 | 38.10% |
| Years of working |  |  |  |  |  |  |
| 1～ | 5 | 62.50% | 6 | 27.27% | 5 | 23.81% |
| 6～ | 2 | 25.00% | 4 | 18.18% | 4 | 19.05% |
| 11～ | 1 | 12.50% | 5 | 22.73% | 5 | 23.81% |
| 21～ | 0 | 0.00% | 7 | 31.82% | 7 | 33.33% |
| Education |  |  |  |  |  |  |
| Junior college | 0 | 0.00% | 1 | 4.55% | 1 | 4.76% |
| Undergraduate | 2 | 25.00% | 3 | 13.64% | 2 | 9.52% |
| Postgraduate | 5 | 62.50% | 16 | 72.73% | 16 | 76.19% |
| PhD | 1 | 12.50% | 2 | 0.00% | 2 | 0.00% |
| Speciality |  |  |  |  |  |  |
| Statistics/Epidemics | 2 | 25.00% | 7 | 31.82% | 7 | 33.33% |
| General Practice/Rehabilitation | 3 | 37.50% | 9 | 40.91% | 8 | 38.10% |
| Health management | 3 | 37.50% | 5 | 22.73% | 5 | 38.10% |
| Other | 0 | 0.00% | 1 | 4.55% | 1 | 4.76% |
| Title |  |  |  |  |  |  |
| Primary | 2 | 25.00% | 1 | 4.55% | 1 | 4.76% |
| Middle | 5 | 62.50% | 13 | 59.09% | 12 | 57.14% |
| High | 1 | 12.50% | 8 | 36.36% | 8 | 38.10% |
| Total | 8 | 100.00% | 22 | 100.00% | 21 | 100.00% |

**Supplementary Table 2:** Comparison Matrix Formulation Guidelines

| **Scale** | **Meaning** |
| --- | --- |
| 1 | $\chi_{i}$ is as important as $\chi_{j}$ |
| 3 | $\chi_{i}$ is slightly more important than $\chi_{j}$ |
| 5 | $\chi_{i}$ is significantly more important than $\chi_{j}$ |
| 7 | $\chi_{i}$ is strongly more important than $\chi_{j}$ |
| 9 | $\chi_{i}$ is extremely more important than $\chi_{j}$ |
| 2,4,6,8 | Represents the intermediate condition of the above two adjacent judgments |
| count backwards | If the importance ratio of $\chi_{i}$ to $\chi_{j}$ is $a_{ij},$then the importance ratio of $\chi_{j}$ to $\chi_{i}$ is $a_{ji}$=1/$a_{ij}$. |
